# Lemmas induce dormancy but help the seed of *Leymus chinensis* to resist drought and salinity conditions in Northeast China

**DOI:** 10.7717/peerj.1485

**Published:** 2016-01-04

**Authors:** Jixiang Lin, Shuai Shao, Na Zhang, Yang Wang, Chunsheng Mu

**Affiliations:** 1 Key Laboratory of Saline-alkali Vegetation Ecology Restoration in Oil Field, Ministry of Education, Northeast Forestry University, Haerbin, Heilongjiang, China; 2 Key Laboratory of Vegetation Ecology of Ministry of Education, Institute of Grassland Science, Northeast Normal University, Changchun, China

**Keywords:** *Leymus chinensis*, Lemmas, Drought, Salinity, Recovery

## Abstract

*Leymus chinensis* is a dominant grass in the Songnen grassland of Northern China. The lower germination caused by the presence of lemmas has proved to be an obstacle for the use of the seeds of this plant by humans. However, it is still unknown if the lemmas have other ecological roles such as resisting drought and saline conditions. Three experiments were designed to investigate the ecological roles of the lemmas in *Leymus chinensis* seeds. The results showed that lemmas significantly improved the amount of water uptake and slowed down the dehydration rate of the seeds under dry conditions. Likewise, the lemmas induced seed dormancy, and removal of the lemmas improved the germination at all temperatures. Although germination percentage of the seeds without lemmas were higher than that of seeds with lemmas under salinity stress, the recovery and total percentage were significantly lower than the seeds with lemmas, especially at 400 mM stress. These results suggest that the lemmas play a vital function in water uptake, dehydration and salt tolerance during the germination stage of the seeds as a response to adverse environmental conditions. Although lemmas showed a dormancy effect, if we want to plant this species in salinity soil in Northeast China, the approach of removing the lemmas by artificial means and improving the seed germination percentage is not feasible.

## Introduction

*Leymus chinensis* is a perennial rhizomatous species of the Poaceae family. It is widely distributed in the eastern region of the Eurasian steppes, the Northern and Eastern parts of the People’s Republic of Mongolia, the Inner Mongolia Plateau of China, and the Northeast China Plains (Lin et al., 2011). This grass has an ecological and economical importance because of its high nutritional content (proteins, minerals and carbohydrates). Due to its high palatability, it is used as fodder for grazing livestock. In addition, it also has a high tolerance to arid and saline-alkaline soils in the Northeast of China (Huang et al., 2002). Due to environmental problems and human activity, such as irrational land reclamation and overgrazing, available grasslands have increasingly been degraded. *Leymus chinensis* is considered as one of the most promising grass species for grassland rehabilitation and restoration in Northern China (Liu & Qi, 2004; Yang, Liu & Zhang, 1995). Therefore, there is a growing demand for *Leymus chinensis* seeds.

The lower germination percentage caused by the seed dormancy of *Leymus chinensis* has proved to be an obstacle for human use to restore deteriorated grasslands. Previous studies have demonstrated that the mechanical resistance of the lemmas played a key role in the dormancy of *Leymus chinensis* seeds. The lemmas offer a high mechanical resistance to the small embryo, which prevents it from absorbing water from surroundings (Liu & Qi, 2004; Ma et al., 2008; Ma et al., 2010). Lemmas have also been reported to impose dormancy in several other species such as *Dodonaea viscosa* and barley (Benech-Arnold et al., 1999; Baskin & Baskin, 1998; Phartyal et al., 2005). Therefore, several research efforts have tried to explore how to remove the lemmas by artificial means and enhance the seed germination percentage of *Leymus chinensis*. However, certain researchers reported that the seed coat was assumed to be beneficial for long-term seed survival in the soil, especially in harsh, dry environments (Chacón & Bustamante, 2001; Hu, Wang & Wu, 2009). In the Songnen grassland of China, *Leymus chinensis* exists as the dominant species. This region has an arid to semi-arid climates, and the soil salinity is high. The lemmas impose dormancy on the seeds; however, it is not known if these structures have other ecological roles, such as adapting the seeds to salinity and drought conditions.

Soil salinization is a major environmental problem throughout the world, and especially in Northeast China. High salinity in the soil always results in both a delay and a reduction of seed germination. In addition, it can cause a complete inhibition of germination if the saline concentration is high enough (Ungar, 1991; Lin et al., 2011; Lin et al., 2014). However, due to the irregular rainfall in this region, the saline concentration in the soil constantly changes. Previous studies focused on fixed stress duration (Khan & Gulzar, 2003; Guo, Shi & Yang, 2009). Infact, seeds in the soil usually face different stress durations and intensities. In addition, the germination recovery test is often used to determine whether the seeds are killed or merely their germination is prevented by the salinity stress (Guan et al., 2009).

In this study, three independent experiments were designed to examine the ecological role (dormancy and salt tolerance) of the lemmas in *Leymus chinensis* seeds. We aimed at determining (1) the effect of lemmas on the water uptake and dehydration of the seeds, (2) the effect of different temperatures on the seed germination (with and without lemmas), and (3) the effect of different salt stress durations on the seed germination (with and without lemmas). A better understanding of the ecological roles of the lemmas should facilitate an effective utilization of this species under harsh environments. Likewise, it would provide opportunities for improving the grassland restoration and management.

## Materials and Methods

### Plant material

**Figure 1:**
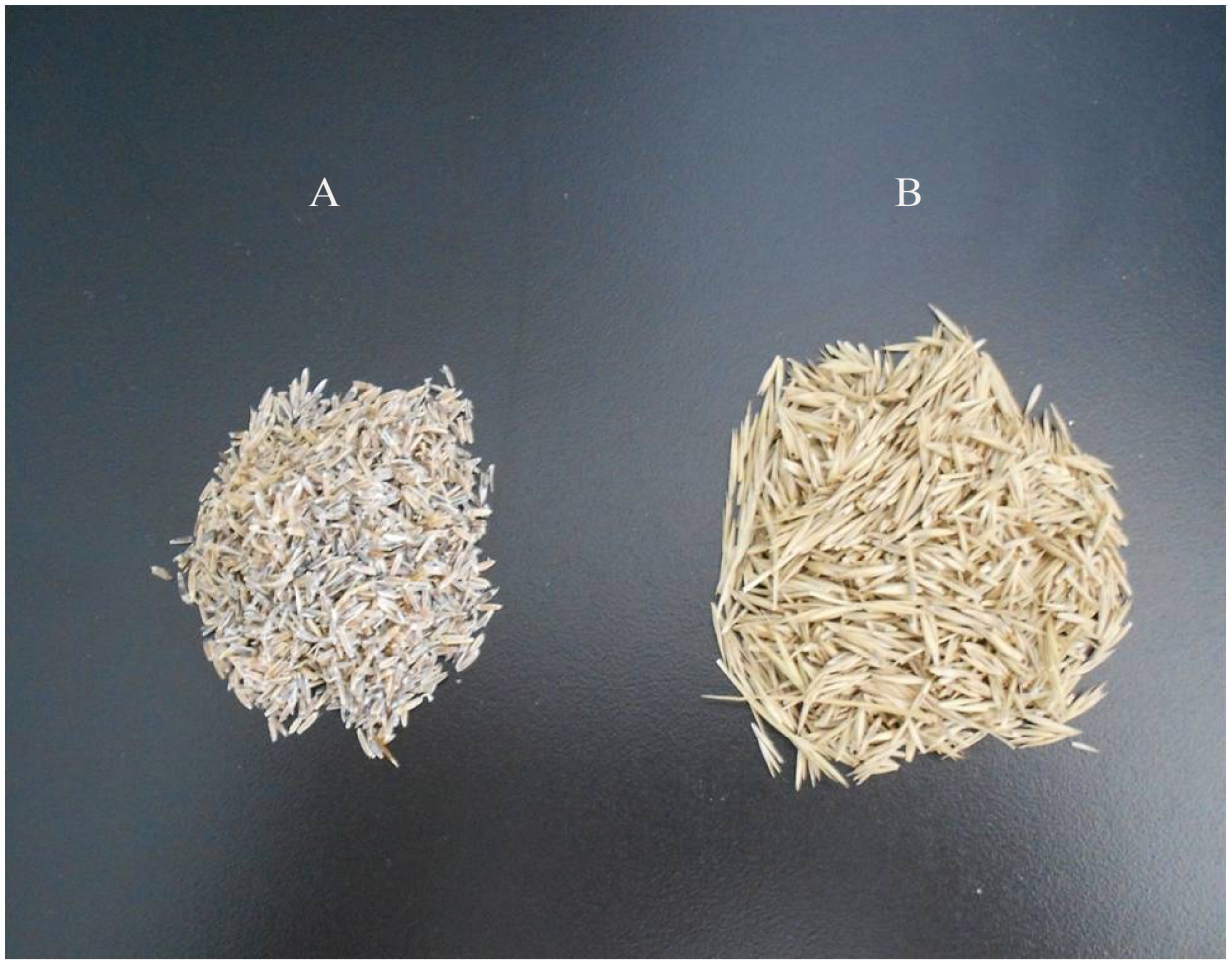
*Leymus chinensis* seeds without lemmas (A) and withlemmas (B).

Mature seeds (Thousand-seed weight is 2.4 g) of *L. chinensis* were collected from the Grassland Ecosystem Field Station, Institute of Grassland Science, Jilin province, China (123°44′E, 44° 44′N). This area is characterized by a semi-arid, continental monsoonal climate. The mean annual precipitation is 370 mm, and the mean annual temperature is 5.5 °C. The seeds were stored in paper bags at 4 °C until further use. In addition, the seeds were surface-sterilized in 0.1% mercury chloride for 10 min and then washed with distilled water and air-dried to avoid fungal infection before being used in our experiments. Then, the seeds were divided into two groups: with lemmas or without lemmas, removed by hand (Fig. 1).

### Experimental design

#### Effects of lemmas on the water uptake and dehydration of the seeds

For each treatment, three replicates of 50 seeds were used. The two groups of seeds (with and without lemmas) were weighed at the beginning of the test. Seeds were placed in 11 cm Petri dishes on two layers of 12.5 cm filter paper moistened with 10 mL of the distilled water and incubated in 25 °C growth chambers. The water uptake was recorded every 1 h during a 24 h-period to determine the amount and rate of water uptake. For the dehydration experiment, seeds with and without lemmas were weighed at the beginning of the test, soaked in water for 24 h, and then placed on a dry filter paper. The seeds were also weighed every 1 h until the seed weight reached the initial weight to calculate the amount of water retention.

#### Effects of temperature regimes on seed germination

Alternating temperature regimes of 20/10, 25/15, 30/20 and 35/25 °C as well as constant temperature regimes of 10, 15, 20, 25 and 30 °C were used. The higher temperatures (20, 25, 30 and 35 °C) coincided with a 12 h photoperiod and the lower temperatures with the 12-h dark period in the growth chambers (HPG-400, Haerbin, China). Seeds were placed in 11 cm Petri dishes on two layers of 12.5 cm filter paper moistened with 10 mL distilled water. Four replicates of 50 seeds in each group (with and without lemmas) were used in this experiment. The germination percentage and rate were recorded every 2 days for 20 d. The germination rate was estimated by using a modified Timson index of germination velocity, ∑*G*∕*t*, where *G* is the percentage of seed germination and *t* is the germination time (Khan & Ungar, 1984). The maximum value obtained in our experiment was 50 (i.e., 1000/20).

#### Effects of salt stress duration on seed germination

In this experiment, five salt concentrations (50, 100, 200, 300 and 400 mM NaCl), and three stress durations (8 d, 16 d and 24 d) were used according to a preliminary test of salt tolerance of the seeds. Four replicates of 50 seeds with and without lemmas were used for each treatment, and distilled water was used as the control. The Petri dishes were placed in growth chambers and maintained at 30/20 °C with a photoperiod of 12 h (Sylvania cool white fluorescent lamps, 200 µmol m^−2^ s^−1^, 400–700 nm, HPG-400, Haerbin, China). The seeds were considered germinated when the radicle emerged. The germination percentage was recorded every day for 8, 16 and 24 d in each treatment. The seeds that did not germinate from all the treatments were then transferred to distilled water to study the recovery percentage of germination, which was also recorded every day during 20 d. Total germination percentage was calculated based on the initial germination percentage and the recovery germination percentage.

### Data analysis

The germination data were arcsine transformed before an analysis of variance (ANOVA). The data were analyzed using SPSS 13.0 (SPSS Inc., Chicago, IL, USA). A two-way ANOVA was used to test the effects of the factors (lemmas and temperature) (lemmas, salinity and stress duration) and their interactions on the germination percentage, germination recovery percentage and total germination percentage. A Tukey’s test was carried out to determine significant differences between the means (*P* < 0.05).

## Results and Discussion

### Effects of the lemmas on the water uptake and dehydration of the seeds

**Figure 2:**
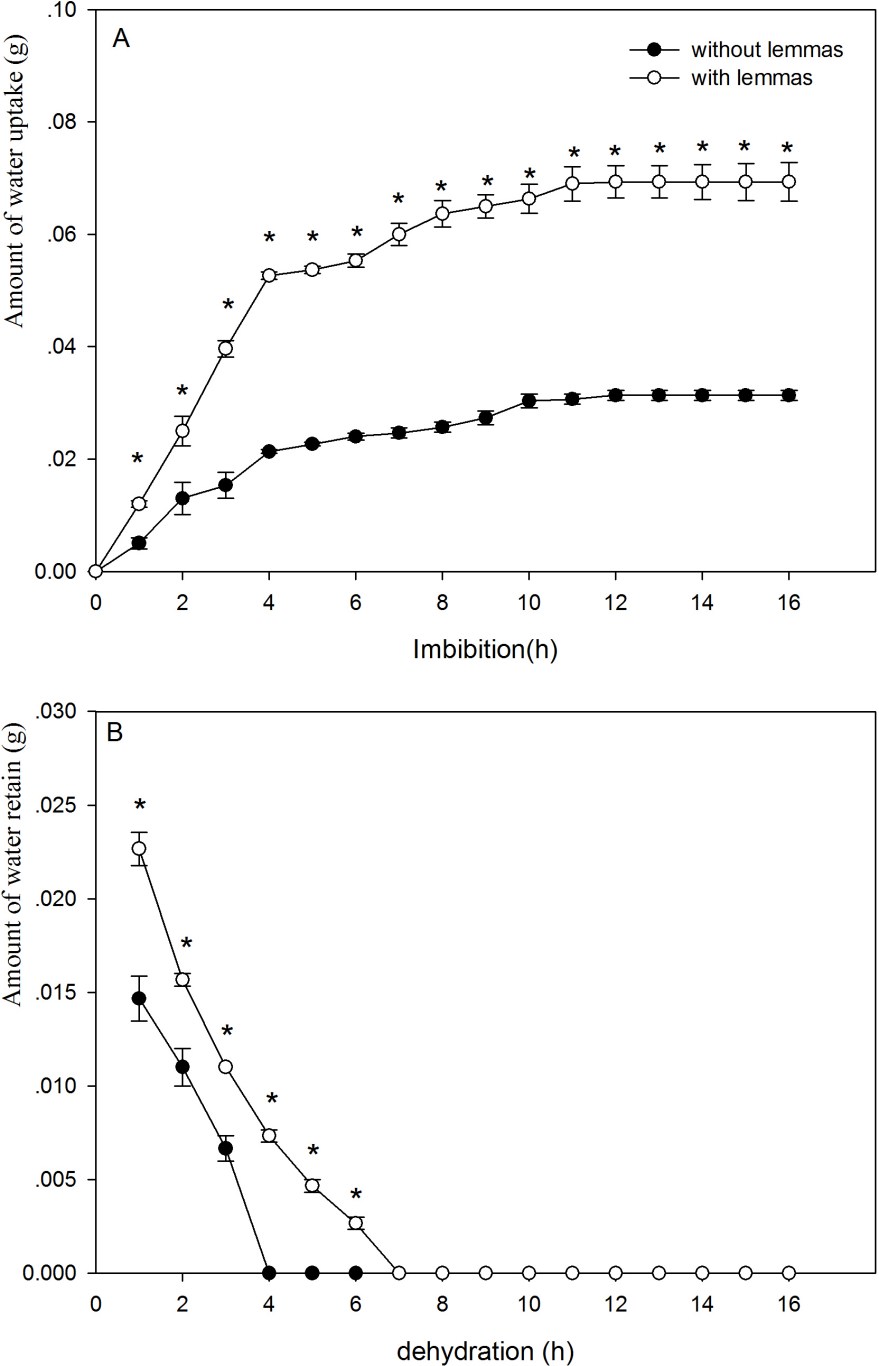
Amount of wateruptake (A) and of water retain in the seeds (B) of with lemmas and withoutlemmas of *Leymus chinensis*. Note: * was significantly different between seeds with lemmas and without lemmas in the same imbibition or dehydration time at 0.05 level. Bars represent mean ± S.E. (*n* = 4).

The presence of the lemmas had a significant effect on the water uptake and dehydration of the seeds (Fig. 2A). The lemmas significantly improved the amount of water uptake of the *Leymus chinensis* seeds. During the initial 4 h, the amount of water uptake of the seeds with lemmas was 2.5 times higher than that of the seeds without lemmas. Likewise, the amount of water uptake of the seeds with lemmas was 2.2 times higher than that of the seeds without lemmas after 24 h (*P* < 0.05). The lemmas also kept the *Leymus chinensis* seeds maintain more water (*P* < 0.05, Fig. 2B). Seeds without lemmas reached the initial water content when dried after 4 h, while it took 7 h in seeds with lemmas.

Above results clearly show that the lemmas affect water uptake and loss of the seeds. The lemmas improved the amount of water uptake and also prolonged the time to complete dehydration of the seeds under drought conditions. Hence, the lemmas might increase the seed surface area, which helps the seed intake water more quickly, and act as a buffer reducing the rate of water uptake (Powell, Oliveira & Mattews, 1986). Based on our results, the lemmas significantly increased the amount of water uptake, especially during the initial 10 h. This characteristic might protect the seeds under dry conditions because the seeds can absorb more water and increase the water use efficiency to resist the drought stress. However, this result is not in agreement with that of Hu, Wang & Wu (2009) for *Hedysarum scoparium*. This discrepancy might be the result of the different plant species, seed size, adaptive mechanism and living environments of the two species. In addition, the dehydration lasted for 7 h to reach the initial weight for the seeds with lemmas but only 4 h without lemmas. This result suggests that the lemmas can prolong dehydration period and allow the seeds to retain enough water to survive under short term dry environments. Similar results have also been reported for other plants such as *Cryptocarya alba* (Chacón & Bustamante, 2001).

### Effects of the temperature regimes on seed germination

**Figure 3:**
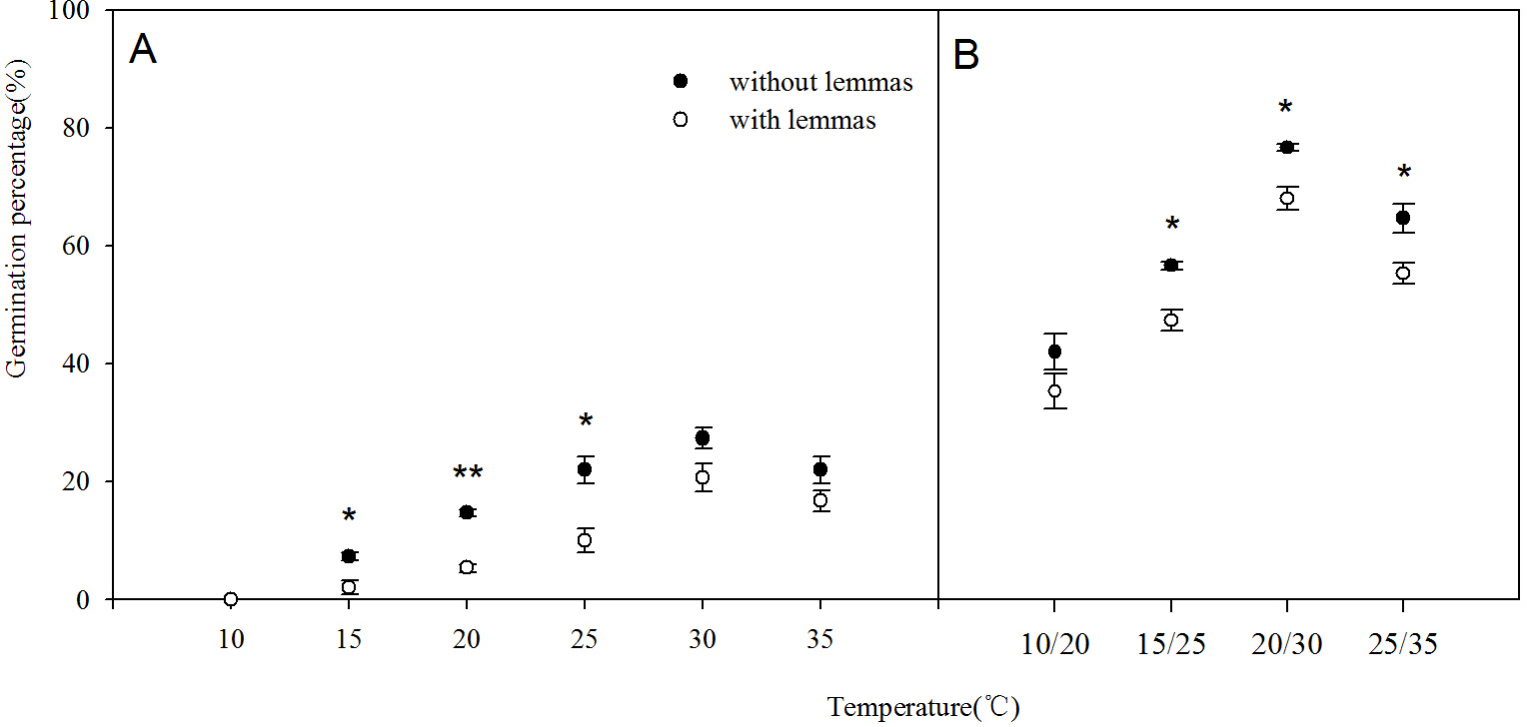
Effects of different temperature regime on seed germination of *Leymus chinensis* (with lemmas and without lemmas). Note: * was significantly different between seeds with lemmas and without lemmas in the same temperature at 0.05 level. Bars represent mean ± S.E. (*n* = 4).

**Figure 4:**
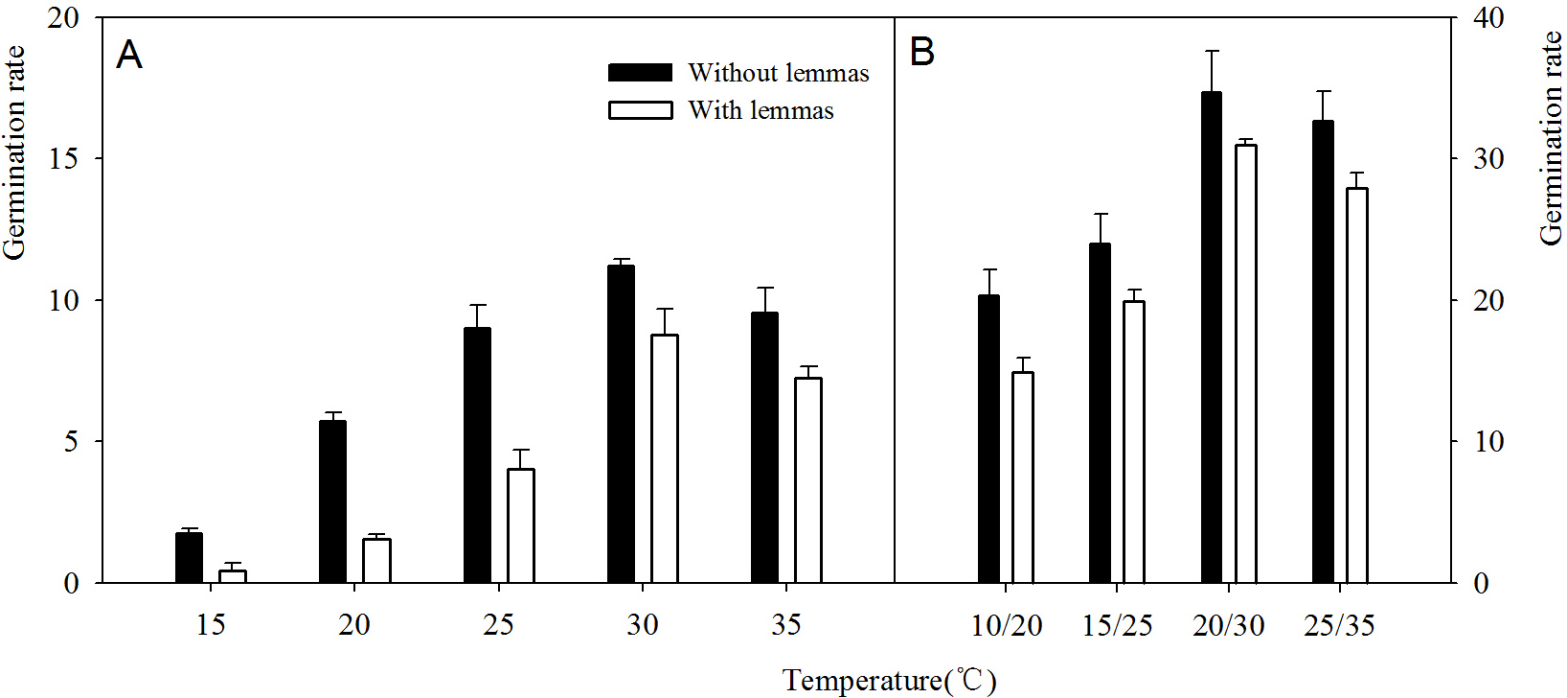
Effects of different temperature regime on germination rate of *Leymus chinensis* seeds (with and without lemmas). Bars represent mean ± S.E. (*n* = 4).

Two-way ANOVA analysis result showed that the germination percentage and rate were both affected by the lemmas and temperatures (Table 1). The germination percentage and germination rate of the seeds without lemmas were both significantly higher than those of the seeds with lemmas in each temperature regime. Under constant temperatures (15–35 °C), the germination percentage of the seeds without lemmas were 5.3%, 9.3%, 12%, 6.7% and 5.4% higher than that for the seeds with lemmas, respectively (Fig. 3). The germination rate was similar to the germination percentage (Fig. 4). In addition, the seed germination percentage and rate were higher in alternating temperatures than that in constant temperatures, indicating alternating temperature regimes are beneficial for seed germination of this species.

**Table 1:** Two-way analyses of variance for the effect of lemmas and temperature on seed germination and germination rate of *Leymus chinensis.*

Independent variable	Germination percentage				Germination rate			
	df	MS	*F*	*P*	df	MS	*F*	*P*
L	1	880.1	82.5	^***^	1	69.9	72.8	^***^
T	9	3,418.3	320.5	^***^	9	77.1	80.7	^***^
L × T	9	7.4	0.69	0.693	9	3.3	3.4	0.027

**Notes.**

L Lemmas T Temperature df Degree of freedom MS Mean Square F F-ratio

*** Factors are significant at *P* < 0.001 level.

These results show that the lemmas induced seed dormancy and decreased the seed germination indexes. Once the seeds were without lemmas, germination percentage and rate improved. The lemmas might impose a mechanical resistance and restrict the gas exchange. In addition, previous studies found that the lemmas contained certain hormones such as abscisic acid that might affect the seed germination (Yi, 1994; Ma et al., 2008; Ma et al., 2010). Other studies have also reported that seed coat and certain structure such as lemmas imposed dormancy on seeds (Robertson et al., 2006). However, this phenomenon has important ecological significance. The seed dormancy caused by the lemmas might be beneficial for *Leymus chinensis* in coping with the dry environments of its native range. The lemmas prevent the rapid desiccation and control the germination process. Germination only occurs after a rainfall, which creates suitable conditions for seedling growth and survival (Bustamante & Grez, 1993).

### Effects of salt stress duration on seed germination

Two-way ANOVA analysis result showed that the germination recovery percentage and total percentage were both significantly affected by the presence/absence of lemmas, stress duration, stress intensity and their interactions. For seed germination percentage, except for the interactions of lemmas and stress intensity, germination percentage was also significantly affected by the other factors and their interactions (Table 2).

**Table 2:** Three-way analyses of variance for the effect of lemmas, stress time and salinity on seed germination index of *Leymus chinensis*.

Independent variable	Germination percentage				Recovery percentage				Total percentage			
	df	MS	*F*	*P*	df	MS	*F*	*P*	df	MS	*F*	*P*
L	1	2,352.0	187.3	^***^	1	7,720.1	823.8	^***^	1	273.9	45.7	^***^
T	2	1,194.5	95.1	^***^	2	3,888.0	414.9	^***^	2	219.4	36.6	^***^
S	5	9,295.9	740.3	^***^	4	1,487.8	158.8	^***^	5	1,232.9	205.5	^***^
L × T	2	49.0	3.9	0.025	2	543.3	58.0	^***^	2	135.8	22.6	^***^
L × S	5	17.2	1.4	0.247	4	35.6	3.8	0.008	5	258.7	43.1	^***^
T × S	10	106.8	8.5	^***^	8	21.4	2.3	^***^	10	32.1	5.4	^***^
L × T × S	10	37.5	3.0	^***^	8	120.3	12.8	^***^	10	53.0	8.8	^***^

**Notes.**

L Lemmas T Temperature S Salinity df Degree of freedom MS Mean Square F F-ratio

*** Factors are significant at *P* < 0.001 level.

**Table 3:** Effects of lemmas, stress time and salinity on germination of *Leymus chinensis*.

Stress days	Salinity	Germination percentage		Recovery percentage		Total germination	
		Without lemmas	With lemmas	Without lemmas	With lemmas	Without lemmas	With lemmas
8d	0	76.7 ± 0.7a	68.0 ± 1.4a	–	–	76.7 ± 0.7a	68.0 ± 1.4a
	50	69.3 ± 0.7b	54.0 ± 2.0b	17.4 ± 2.0a	52.2 ± 1.3a	74.7 ± 0.7a	78.0 ± 1.2b
	100	60.7 ± 1.8c	46.0 ± 3.5c	23.9 ± 0.8b	55.6 ± 1.9a	70.0 ± 1.2b	76.0 ± 2.0b
	200	34.0 ± 2.0d	28.7 ± 3.7d	28.2 ± 1.2c	62.8 ± 1.5b	52.7 ± 0.7c	73.3 ± 2.4b
	300	24.7 ± 1.8e	12.7 ± 1.8e	37.9 ± 1.5d	60.3 ± 1.0b	53.3 ± 0.7c	65.3 ± 0.7a
	400	14.0 ± 1.2f	2.7 ± 0.7f	45.0 ± 1.0e	63.0 ± 0.9b	52.7 ± 1.3c	64.0 ± 1.2a
16d	50	69.3 ± 0.7ab	65.3 ± 2.9a	21.8 ± 2.5a	25.1 ± 0.9a	76.0 ± 1.2a	74.0 ± 2.3b
	100	66.0 ± 2.0b	56.0 ± 2.3b	27.4 ± 3.7ab	39.1 ± 2.1b	75.3 ± 1.3a	73.3 ± 0.7b
	200	56.7 ± 3.5c	38.0 ± 2.0c	30.7 ± 1.1bc	40.8 ± 2.1b	70.0 ± 2.3b	63.3 ± 1.3a
	300	38.0 ± 5.0d	24.0 ± 3.1d	31.9 ± 2.5bc	52.6 ± 2.8c	58.0 ± 2.0c	64.0 ± 2.0a
	400	20.7 ± 1.8e	12.7 ± 1.3e	36.1 ± 1.5c	61.1 ± 0.9d	49.3 ± 0.7d	66.0 ± 1.2a
24d	50	73.3 ± 0.7a	64.7 ± 0.7a	7.5 ± 0.1a	13.2 ± 1.8a	75.3 ± 0.7a	69.3 ± 1.2a
	100	65.3 ± 1.3b	58.0 ± 2.0b	9.7 ± 2.1ab	20.7 ± 1.3b	68.7 ± 1.8b	66.7 ± 3.1a
	200	54.7 ± 2.9c	56.0 ± 1.2c	16.1 ± 0.7bc	25.9 ± 2.1b	62.0 ± 2.3c	67.3 ± 3.0a
	300	40.7 ± 0.7d	29.3 ± 0.7d	20.2 ± 1.8cd	35.0 ± 2.7c	52.7 ± 0.7d	54.0 ± 4.0b
	400	22.0 ± 3.1e	15.3 ± 1.3e	24.6 ± 3.7d	48.8 ± 1.3d	41.3 ± 1.8e	56.7 ± 2.3b

**Notes.**

Different letters indicate significant differences between stress treatments *P* < 0.05.

The seed germination percentages (with and without lemmas) decreased (*P* < 0.05) but the recovery percentage increased with higher saline concentrations. The maximum recovery percentage was obtained with a salinity stress of 400 mM (Table 3). In addition, the recovery percentage decreased in treatments with longer stress durations. Compared with the 8 d stress, the recovery percentages of the seed without lemmas and with lemmas were 20.4% and 14.2% lower at 400 mM under 24 d stress. Hence, the total germination percentage was the lowest with 24 d of stress.

Although the germination percentage of the seeds without lemmas was higher than that of the seeds with lemmas under salinity stress, the recovery percentage was significantly lower in the seeds without lemmas than that of the seeds with lemmas, especially at 400 mM salinity stress. The percentage reductions were 18%, 30% and 24%, respectively. The total germination percentages of the seeds without lemmas were also lower than those of the seeds with lemmas.

Our results indicate that the recovery percentage in *L. chinensis* seeds (with and without lemmas) showed increasing trends with salinity increments in all the treatment groups. This outcome constitutes an adaptive strategy of seed germination to higher salinity stress. Thus, the seeds that did not germinate remained in a state of dormancy to escape from the rigorous environments such as higher salinities (Debez et al., 2004). This phenomenon indicates that a high salinity only postponed the germination process for many *L. chinensis* seeds but did not cause them to lose viability.

Therefore, the lemmas of the *Leymus chinensis* seeds played an important role in resisting the salt stress, especially at higher salt concentrations. Although the germination percentage of the seeds with lemmas were lower than that of the seeds without lemmas under salinity stress, the recovery percentage and total germination percentage were higher for seeds with lemmas than that of seeds without lemmas. The reasons maybe the protective effects of the lemmas on the seeds, lemmas reduced the intake of salt, and the effect of ion toxicity on the seed was alleviated to some extent. The hormones within the seeds might also play a role in resisting the salt stress. This aspect needs to be further investigated. In addition, field experiment is also very important to test the results in lab conditions, which also needs further research.

## Conclusion

In summary, this study clearly showed the ecological role (dormancy and salt tolerance) of lemmas in *Leymus chinensis* seeds. As an important structure of the *Leymus chinensis* seeds, the lemmas played a vital function in the water uptake, dehydration and salt tolerance during the germination of the seeds in adverse environments (such as dry-wet alternating habitats and other stress conditions). The dormancy effect imposed by the lemmas proved to be beneficial for this species when planted under the salt-alkaline soil conditions of Northeast China. Hence, the approach of removing the lemmas by artificial means to improve the seed germination percentage is not feasible under these conditions.

## Supplemental Information

10.7717/peerj.1485/supp-1 Supplemental Information 1 Raw Data—Effect of lemmas on the water uptake and dehydration of the seeds

10.7717/peerj.1485/supp-2 Supplemental Information 2 Raw Data—Effect of temperature regimes on seed (with and without lemmas) germination

10.7717/peerj.1485/supp-3 Supplemental Information 3 Raw Data—Effect of stresstime of salt stress on seed (with and without lemmas) germination.

## Additional Information and Declarations

### Data Availability

The following information was supplied regarding data availability: The research in this article did not generate any raw data.
